# Author Response: Radiotherapy Increases aMMP-8-Levels and Neutrophil/Lymphocyte Ratio Rapidly in Head and Neck Cancer Patients: A Pilot Study

**DOI:** 10.1177/10732748231187282

**Published:** 2023-08-14

**Authors:** Ella Brandt, Mutlu Keskin, Taina Tervahartiala, Mustafa Yılmaz, İlknur Harmankaya, Didem Karaçetin, Turgut İpek, Ulvi Kahraman Gürsoy, Shipra Gupta, Ismo T. Räisänen, Jaana Hagström, Jaana Rautava, Timo Sorsa

**Affiliations:** 1 Department of Oral and Maxillofacial Diseases, 3835 University of Helsinki and Helsinki University Hospital, Helsinki, Finland; 2 Oral and Dental Health Department, 187458 Altınbaş University, İstanbul, Turkey; 3 Department of Periodontology, Faculty of Dentistry, 3835 Biruni University, Istanbul, Turkey; 4 Department of Radiation Oncology, 622463 Başakşehir Çam and Sakura City Hospital, Istanbul, Turkey; 5 Department of General Surgery, Faculty of Medicine, 187458 Altınbaş University, Istanbul, Turkey; 6 Department of Periodontology, Institute of Dentistry, 8058 University of Turku, Turku, Finland; 7 Unit of Periodontology, Oral Health Sciences Centre, 29751 Post Graduate Institute of Medical Education and Research, Chandigarh, India; 8 Department of Pathology, 3835 Helsinki University Hospital & HUSLAB, Helsinki; 9 Department of Oral Pathology and Radiology, 8058 University of Turku, Turku, Finland; 10 Department of Medicine and Dental Medicine, Karolinska Institute, Stockholm, Sweden

We thank the writer for the interest and constructive comments considering our study. As this is an observational pilot study, we have wished to emphasize the limitations of this study and avoid any definite conclusions. The results must be confirmed with larger studies and ideally distinguish the different patient groups and treatments. The findings of this study encouraged us to proceed in this topic and that is what we are currently working on. Therefore, we are very grateful for the suggestions and comments in the letter.

It is true that there are variations on tumors, locations, treatments, and radiation fields to some extent but even considering this, our results for each patient were quite consistent supporting the true nature of our concern for periodontal tissues of cancer patients receiving radiotherapy on the head and neck region. This manuscript wishes to bring into the knowledge the new information of the possibility to utilize easy access biomarkers, favorably to be used chair-/bedside, detecting possible HNC side effects. Based on the findings of this study, as patients received radiotherapy to the larynx/oropharynx/nasopharynx/parotitis, we suggest that the primary radiation in HNC treatment doesn’t need to be directly in the gums or jaws to have impact on periodontal tissues and increase risk for periodontitis. Advantage with the aMMP-8 oral rinse test is that it is not site-specific, and if the test turns out to be useful in HNC radiotherapy patients, it could be used regardless of the location of primary tumor. Early detection of periodontitis risk helps both patient and professional to react before experiencing tissue destruction.

We would like to point out that periodontal examination procedure was discussed in the article and as in proper periodontal examination all teeth surfaces are examined. As a hospital routine protocol, periodontal examination was done after RT, but that data has not been used in this study because in this manuscript we wanted to focus on the first weeks of radiotherapy and the early effect of RT on biomarkers.

All patients had periodontitis, so receiver operating curve (ROC)-analysis would not have been suitable here in the absence of healthy controls. As the data consisted of before and after measurements of the patients, the paired samples t-test was utilized to test the potential effects of RT in HNC patients. The suggestion with utilization of ROC-analysis will be gratefully noted in the statistics of the following study designs.

